# Single-Cell-State Culture of Human Pluripotent Stem Cells Increases Transfection Efficiency

**DOI:** 10.1089/biores.2016.0009

**Published:** 2016-05-01

**Authors:** Takenobu Nii, Hiroshi Kohara, Tomotoshi Marumoto, Tetsushi Sakuma, Takashi Yamamoto, Kenzaburo Tani

**Affiliations:** ^1^ Division of Molecular and Clinical Genetics, Medical Institute of Bioregulation, Kyushu University, Fukuoka, Japan.; ^2^ Project Division of ALA Advanced Medical Research, The Institute of Medical Science, The University of Tokyo, Tokyo, Japan.; ^3^ Department of Advanced Molecular and Cell Therapy, Kyushu University Hospital, Fukuoka, Japan.; ^4^ Department of Mathematical and Life Sciences, Graduate School of Science, Hiroshima University, Hiroshima, Japan.

**Keywords:** gene expression, gene transfer, stem cells

## Abstract

Efficient gene transfer into human pluripotent stem cells (hPSCs) holds great promise for regenerative medicine and pharmaceutical development. In the past decade, various methods were developed for gene transfer into hPSCs; however, hPSCs form tightly packed colonies, making gene transfer difficult. In this study, we established a stable culture method of hPSCs at a single-cell state to reduce cell density and investigated gene transfection efficiency followed by gene editing efficiency. hPSCs cultured in a single-cell state were transfected using nonliposomal transfection reagents with plasmid DNA or mRNA encoding enhanced green fluorescent protein. We found that most cells (DNA > 90%; mRNA > 99%) were transfected without the loss of undifferentiated PSC marker expression or pluripotency. Moreover, we demonstrated an efficient gene editing method using transcription activator-like effector nucleases (TALENs) targeting the adenomatous polyposis coli (*APC*) gene. Our new method may improve hPSC gene transfer techniques, thus facilitating their use for human regenerative medicine.

## Introduction

Human pluripotent stem cells (hPSCs), such as human embryonic stem cells (hESCs) and human-induced pluripotent stem cells (hiPSCs), have the potential to self-renew indefinitely and differentiate into various cell types. hPSCs can differentiate into various stem or progenitor cell populations used for regenerative medicine and drug development. The newly developed genome editing technology has advanced the use of hPSCs for such purposes. However, to fully utilize hPSCs to achieve this goal, more efficient gene transfer methods under defined conditions are required.

Several published methods are used for gene transfer into hPSCs, such as transfection,^1–4^ electroporation,^2,3,5^ nucleofection,^3,6^ and viral transduction.^2,7,8^ Braam et al. improved the efficiency of gene transfer into hESCs by reducing cell density under feeder cell-free conditions.^2^ However, their protocol required trypsinization for initial cell adaptation to low-density culture. Most of the cells might die after this treatment,^9^ and surviving cells might display altered growth characteristics.^10^ Moreover, in these experiments, cells were cultured on Matrigel-coated dishes in the mouse embryonic fibroblast (MEF)-conditioned medium that contained unknown cell culture factors. Such unknown factors are likely to impede the reproducibility of the culture.

Recently, a single cell passage supported hPSC culture method was developed. This system utilizes Pluripro MATRIX, a fully defined mixture of highly purified human sera proteins.^11^ Interestingly, when small clumps of hPSCs were plated on this MATRIX, they became more dispersed and did not form tightly packed colonies, indicating that these culture conditions provided a stable, low–cell density culture system for hPSCs.

Development of efficient gene editing methods, such as zinc-finger nucleases (ZFNs), transcription activator-like effector nucleases (TALENs), and clustered regularly interspaced short palindromic repeat (CRISPR)/CRISPR-associated nuclease 9 (Cas9), for use in hPSCs, holds great promise in the fields of basic and clinical research.^12–18^ Among these methods, TALENs are more efficient and safer for use in hPSCs to achieve specific gene editing, as ZFNs had a low gene editing efficiency and CRISPR/Cas9 was accompanied by more severe off-target effects than TALENs.^19–22^ Electroporation is a widely used transfection method for hPSC genome editing^12–18^; however, this method results in reduced cell viability and gene editing efficiency. In this study, we demonstrate highly efficient and simple DNA and mRNA transfection methods in hPSCs under defined conditions. We then use this method in conjunction with TALENs to demonstrate efficient gene editing in hPSCs.

## Materials and Methods

### hPSC culture

The hESC lines, H1 and H9, and hiPSC lines, 253G4 and 4A (HiPS-RIKEN-4A), were maintained in the human ESC medium as previously described.^23^ The hESC lines (H1 and H9) were purchased from WiCell Research Institute (WI, United States). The cell lines 253G4 and 4A were kindly provided by Prof. Shinya Yamanaka (Kyoto University, Kyoto, Japan) and Dr. Yukio Nakamura (RIKEN BioResource Center, Tsukuba, Japan), respectively. All hPSC lines were transferred to feeder-free conditions. The hPSC colonies were detached from the feeder cells by CTK solution (0.1% collagenase IV, 0.25% trypsin, 20% KSR, and 1 mM CaCl_2_ in phosphate-buffered saline [PBS]). They were also passed through a 40-μm cell strainer to remove feeder cells, and the colonies remaining on the cell strainer were plated on vitronectin-coated (VTN; Life Technologies), Matrigel-coated (BD Matrigel hESC-qualified Matrix; BD Biosciences) or iMatrix-511-coated (nippi, Inc.) dishes in StemMACS™ iPS-Brew XF (Miltenyi Biotec) or Pluripro MATRIX-coated (CELL guidance systems) dishes in Pluripro media (CELL guidance systems). For single-cell-state culture, hPSC colonies were detached using Accutase (Innovative Cell Technologies) and were plated on VTN-, Matrigel-, or iMatrix-511-coated dishes in StemMACS iPS-Brew XF or Pluripro MATRIX-coated dishes in Pluripro media with 10 μM Y-27632 (Nacalai Tesque).

### Plasmid DNA construction and transfection

Plasmid DNA, CSII-EF-EGFP, was constructed from CSII-EF-RfA, which was a kind gift from Dr. Hiroyuki Miyoshi (RIKEN BioResource Center, Tsukuba, Japan). TALEN plasmid DNA driven by the human elongation factor 1-alpha 1 (EF1α) promoter was constructed as described previously^24^ with some modifications. Briefly, DNA-binding modules were assembled with the two-step Golden Gate assembly method using the Platinum Gate TALEN Kit (Addgene). The CMV promoter of ptCMV-136/63-VR vector was replaced with the EF1α promoter. Dissociated hPSCs were seeded at a density of 1.2 × 10^5^/well in MATRIX-coated 12-well plates and cultured for 24 h. Both 1 μg of plasmid DNA and 4 μL of FuGENE HD (Promega) were diluted in 100 μL of Opti-MEM I Reduced Serum Media (Life Technologies) and incubated for 15 min at room temperature. This mixture was transferred to one well of a 12-well plate. Twenty-four hours post-transfection, cells were analyzed for enhanced green fluorescent protein (eGFP) expression to determine transfection efficiency. GeneJammer (Agilent Technologies), X-tremeGENE HP (Roche), and Lipofectamine 2000 (Invitrogen) were used following the manufacturers' instructions. Transfected cells were observed under a fluorescence microscope (BZ-9000; Keyence) and analyzed using BZ-Analyzer software (Keyence).

### mRNA synthesis and transfection

We synthesized mRNA using *in vitro* transcription (IVT) reactions and eGFP polymerase chain reaction (PCR) amplicon or the same TALEN plasmid DNA templates using the mScript mRNA Production System (Epicentre Biotechnologies) according to the manufacturer's protocol. The eGFP IVT template was synthesized using the following primers: sense: 5′-ggatcctaatacgactcactatagggaacagccaccatggtgagcaagggcgagga-3′, antisense: 5′-ttacttgtacagctcgtcca-3′. Dissociated hPSCs were seeded at a density of 1.2 × 10^5^ cells/well in MATRIX-coated 12-well plates for 24 h. mRNA transfections were carried out with a TransIT-mRNA Transfection Kit (Mirus Bio). One microliter of mRNA Boost Reagent, 1 μL of TransIT-mRNA Reagent, and 0.5 μg of mRNA were diluted in 100 μL of Opti-MEM I Reduced Serum Media (Life Technologies) and incubated for 3 min at room temperature. This mixture was transferred to one well of a 12-well plate. Twenty-four hours post-transfection, cells were analyzed for eGFP expression to determine transfection efficiency. Transfected cells were observed by a fluorescence microscope (BZ-9000; Keyence) and analyzed using BZ-Analyzer software (Keyence).

### Electroporation

Electroporation was conducted using the Neon Transfection System (Invitrogen). For electroporation, 1 μg DNA and 1.2 × 10^5^ dissociated hPSCs were mixed in 10 μL resuspension buffer R. Electroporation parameters were as follows: pulse voltage, 1200 V; pulse width, 10 msec; and pulse number, 3. Cells were then plated into VTN-coated 24-well plates in StemMACS iPS-Brew XF supplemented with 10 μM of Y-27632.

### Flow cytometry

Twenty-four hours post-transfection, cells were harvested using Accutase, and analyzed for expression of eGFP by flow cytometry (FCM). For stem cell characterization, hPSCs were fixed in 4% PFA/PBS, blocked with staining buffer (2% FBS/PBS), and then incubated with an antibody against SSEA4 (BD Pharmingen) and NANOG (BD Pharmingen). The cells were detected on a BD FACS Verse flow cytometer (Becton Dickinson), followed by analysis using FlowJo software (Tomy Digital Biology).

### Immunocytochemistry

Immunocytochemistry was conducted as previously described.^25^ Antibodies against NANOG (R&D Systems), OCT3/4 (Santa Cruz), and TRA-1-60 (Santa Cruz Biotechnology) were used. Cells were observed under a fluorescence microscope (BZ-9000; Keyence) and analyzed using BZ-Analyzer software (Keyence).

### Teratoma formation

One million eGFP-transfected cells cultured on MATRIX-coated dishes were centrifuged, and the pellet was resuspended in PBS to a total volume of 50 μL. The cell mixture was combined with 50 μL undiluted cold BD Matrigel Matrix Phenol Red-Free (BD Biosciences) immediately before transplantation. The ROCK inhibitor Y-27632 was added to the cell mixture to a final concentration of 10 μM. NOD.Cg-Prkdcscid Il2rgtm1Sug/Jic (NOG) mice (CIEA, Japan) were used for transplantation. The cell–Matrigel-Y-27632 mixture was injected into the muscle of the right hind leg of the anesthetized mice. After 8 weeks post-transplantation, teratomas were surgically removed, fixed in 4% PFA, embedded in paraffin, sectioned, and stained with hematoxylin and eosin.

### Karyotype assessment

Karyotype analysis of eGFP-encoding plasmid DNA- or mRNA-transfected hESC line (H9) was performed at Chromocenter, Inc. Chromosomes were prepared using standard protocols and G banded with trypsin and stained with Giemsa. For each culture, 20 metaphase spreads were examined.

### Cel-1 assay

The Cel-1 assay was carried out as described previously.^26^ Briefly, 2 days after transfection, cells were collected and the genomic DNA was extracted and used for genomic PCR. PCR was carried out using AccuPrime Taq DNA Polymerase (Invitrogen) with primers described previously.^27^ The products were analyzed by electrophoresis in agarose gels and ethidium bromide staining. The observed ratio of the cleavage product to the parenteral band was determined by ImageJ software, and the gene modification level was estimated.^26^

### Sequence verification of NHEJ-mediated indel mutations

Genomic DNA was extracted 2 days after the last TALEN transfection using the DNeasy Blood & Tissue Kit (Qiagen). Genomic regions flanking the TALEN target sites were PCR amplified by AccuPrime Taq DNA Polymerase with primers described previously.^27^ Purified PCR products were cloned into pGEM-T Easy (Promega) and transformed into JM109 competent cells. Plasmid DNA was isolated for multiple colonies from each transformation and was sequenced using the T7 promoter primer (5′-TAATACGACTCACTATAGG-3′) and BigDye Terminator v3.1 Cycle Sequencing Kit (Life Technologies).

### Statistical analysis

Differences among groups were analyzed for statistical significance with GraphPad Prism 5 software (GraphPad Software) using a Student's *t*-test or ANOVA followed by Tukey's *post hoc* test. A *p* ≤ 0.05 was considered statistically significant.

## Results

### Increased hPSC transfection efficiency using single-cell-state cultures

Transfection efficiency depends on cell density, where efficiency is higher at low cell density than high cell density.^2,28^ hPSC density is remarkably higher than other cell types because hPSCs form tightly packed colonies.^29–31^ Therefore, the transfection efficiency of hPSCs is usually quite low. We found that hPSC colonies cultured without feeder cells were only transfected in marginal regions, which were areas with relatively lower cell density than central colony regions (Supplementary Fig. S1A, B). The calculated percent transfection for DNA and mRNA was 8.75% ± 1.28% (Supplementary Fig. S1C) and 13.9% ± 4.23% (Supplementary Fig. S1D), respectively.

We dissociated hPSCs cultured on VTN or MEFs and passaged cells to several coating dishes for single-cell-state hPSC culture. We tested four commercially available coating substrates, such as VTN, Matrigel, iMatrix-511, and MATRIX. To examine our hypothesis, hPSCs cultured in a single-cell state were transfected with plasmid DNA and mRNA encoding eGFP.

For transfection of eGFP-encoding plasmid DNA, we used several commercial transfection reagents, such as FuGENE HD, GeneJammer, X-tremeGENE HP, and Lipofectamine 2000. FuGENE HD was the most efficient DNA transfection reagent examined (data not shown).

**FIG. 1.**
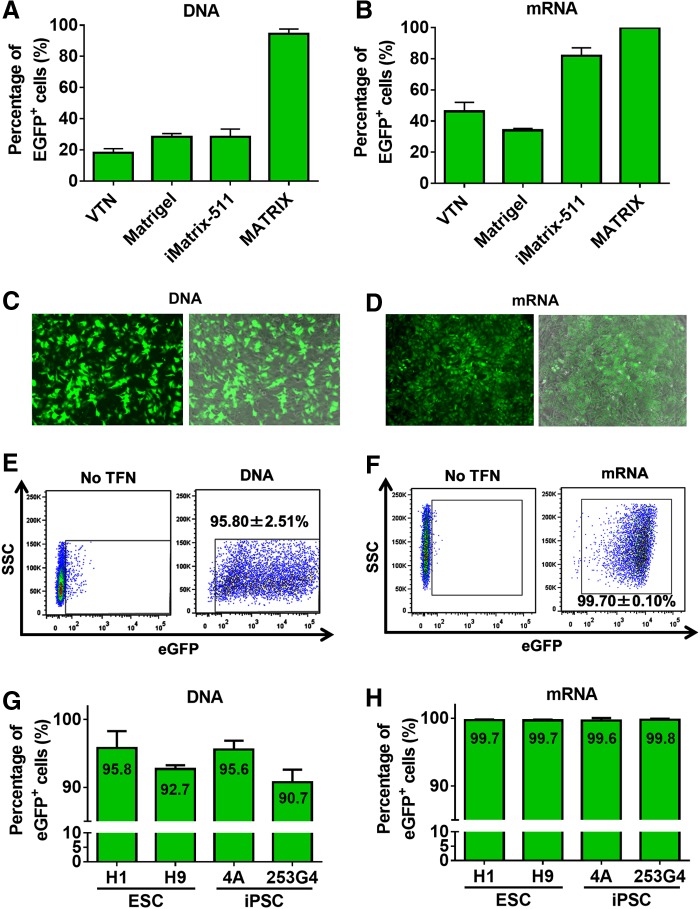

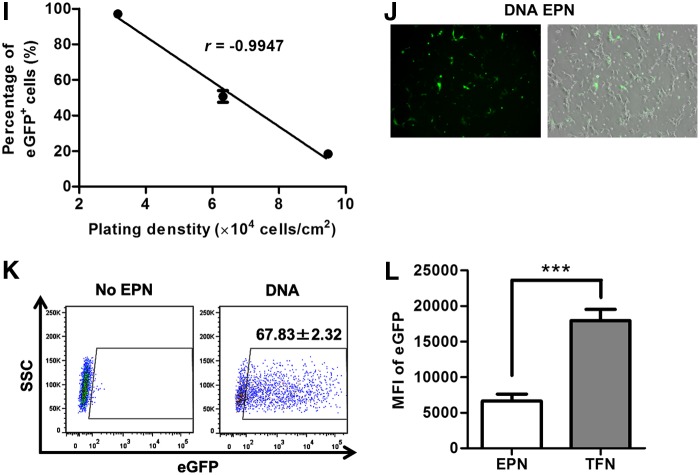
DNA and mRNA transfection of hPSCs under single-state culture. **(A, B)** The gene transfection efficiency of eGFP-encoding DNA **(A)** and mRNA **(B)** for hPSCs cultured under a different culture system. Images of eGFP-encoding DNA-transfected **(C)** and mRNA**-**transfected **(D)** hPSC colonies cultured on MATRIX-coated plates. FCM analysis of eGFP-encoding DNA**-**transfected **(E)** and mRNA**-**transfected **(F)** cells cultured on MATRIX-coated plates. Data are shown as the mean ± SD (*n* = 3). DNA **(G)** or mRNA **(H)** eGFP expression constructs were transfected into hESC lines (H1 and H9) and hiPSC lines (4A and 253G4). Data are shown as the mean ± SD (*n* = 3). **(I)** The relationship between DNA transfection efficiency and plating density. Data are shown as the mean ± SD (*n* = 3). **(J)** Images of eGFP-encoding plasmid DNA electroporated hPSCs cultured on VTN-coated plates. **(K)** FCM analysis of eGFP-expressing plasmid DNA electroporated cells. **(L)** Comparison of eGFP expression levels (MFI) after hPSC TFN of eGFP-encoding DNA vs EPN of eGFP-encoding DNA. Data are shown as the mean ± SD (*n* = 3). ***p < 0.005. eGFP, enhanced green fluorescent protein; EPN, electroporation; FCM, flow cytometry; hESCs, human embryonic stem cells; hiPSCs, human-induced pluripotent stem cells; hPSCs, human pluripotent stem cells; MFI, mean fluorescence intensity; TFN, transfection; VTN, vitronectin.

For eGFP mRNA transfection, we used the TransIT-mRNA Transfection Kit. Twenty-four hours post-transfection, we analyzed the proportion of eGFP^+^ cells by FCM analysis. The proportion of eGFP^+^ cells cultured on VTN-coated dish considerably increased in single-cell-state cultures (DNA: from 8.75% ± 1.28% to 18.13% ± 2.63%; Supplementary Fig. S1C and Fig. 1A, mRNA: from 13.90% ± 4.23% to 46.17% ± 5.90%; Supplementary Fig. S1D and Fig. 1B). Moreover, we demonstrated that the most appropriate coating substrate was MATRIX (DNA: 95.80% ± 2.51%; Fig. 1A, mRNA: 99.70% ± 0.10%; Fig. 1B). The DNA-transfected cells, but not mRNA, showed a significant amount of heterogeneity in their expression levels (Fig. 1C–F and Supplementary Videos 1 and 2). Similar results were obtained using another hESC line (H9) and two hiPSC lines (4A and 253G4) (Fig. 1G, H). Moreover, most of the cells were viable (control: 93.10% ± 0.40%, DNA: 83.40% ± 2.03%, mRNA: 86.71% ± 0.19%, Supplementary Fig. S2D). Our results also demonstrate an inverse correlation between cell density and transfection efficiency (Fig. 1I). eGFP expression was detected by fluorescence microscopy until day 7 post-DNA transfection (data not shown). We performed an immunocytochemical analysis using antibodies against TRA-1-60, NANOG, and OCT3/4 to examine the ability of hPSCs to maintain an undifferentiated state when cultured on MATRIX (Supplementary Fig. S3).

Electroporation is one of the most efficient hPSC gene transfer methods.^5^ Our results showed a transfection efficiency of ∼70% using this method, which was consistent with previous reports^5^ (Fig. 1J, K). The mean fluorescence intensity was approximately threefold higher than that in cells transfected by electroporation (electroporation [EPN]: 6631 ± 992; transfection [TFN]: 17,933 ± 1595, Fig. 1L). These results indicated that single-cell-state hPSC culture improved transfection efficiency.

### Improved transfection efficiency without differentiation or loss of pluripotency in single-cell-state hPSC cultures

**FIG. 2.**
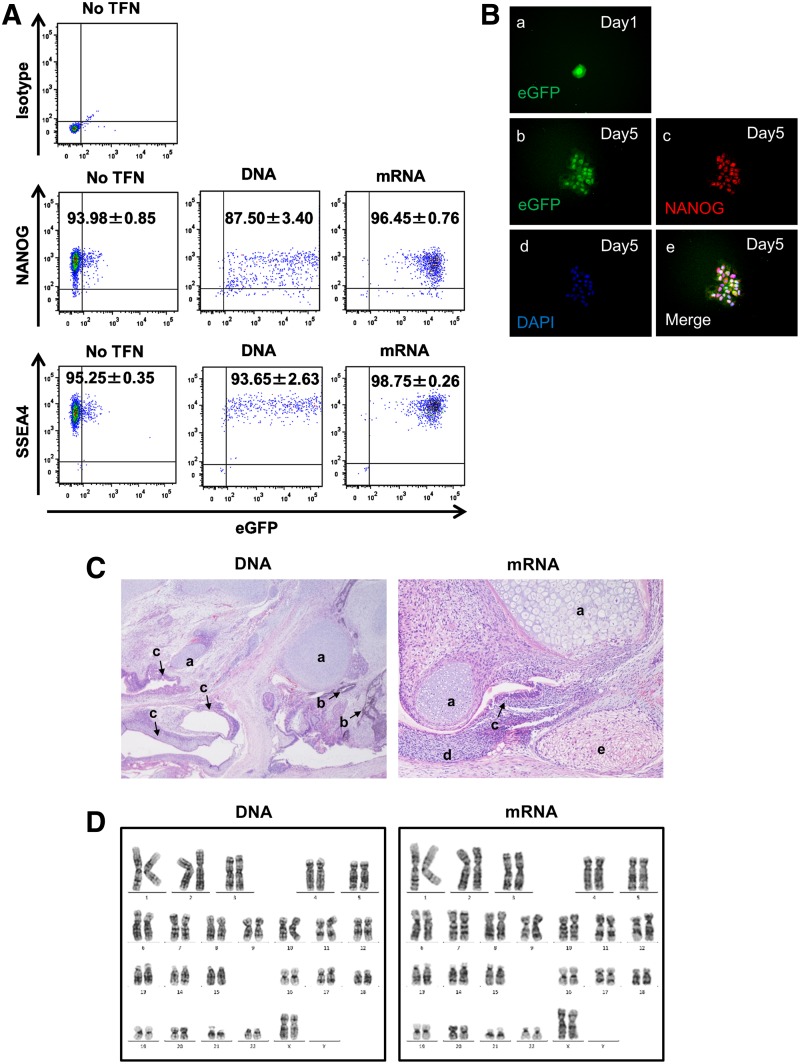
Transfected hPSCs maintain pluripotency and an undifferentiated state. **(A)** DNA- or mRNA-transfected hPSCs cultured on MATRIX-coated plates expressed the undifferentiated PSC markers NANOG and SSEA4. Data are shown as the mean ± SD (*n* = 3). **(B)** DNA-transfected hPSCs expressed NANOG. One day after transfection, DNA-transfected hPSCs were replated on VTN-coated plates (a) and cultured for 4 days (b–e). The expression of NANOG was determined by immunocytochemistry. (b–e) hPSC colony expressing eGFP (b) and NANOG (c). Nuclei stained with DAPI (d). Merged channels (e). **(C)** Teratoma formation of eGFP-encoding DNA- and mRNA-transfected hPSCs. Hematoxylin and eosin staining of the teratoma. (a) Cartilage; mesoderm, (b) melanin-producing cells; ectoderm, (c) gastrointestinal tract-like luminal epithelium; endoderm, (d) mesenchyme; mesoderm, (e) keratinizing epithelium; ectoderm. **(D)** Karyogram of eGFP-encoding DNA and mRNA transfected H9 demonstrating a normal diploid karyotype.

To test whether transfection using this method affected the stemness of hPSCs, we examined SSEA4 and NANOG expression in eGFP-transfected cells by FCM analysis. The percentage of both SSEA4^+^ and NANOG^+^ cells was greater than 90% (Fig. 2A). Twenty-four hours post-DNA transfection, we passaged the cells onto VTN-coated dishes. The cells formed colonies without losing either NANOG or eGFP expression up to 4 days after passage (Fig. 2B). Transplantation of eGFP-transfected cells into immunodeficient mice led to the formation of teratomas (Fig. 2C). Moreover, eGFP-transfected hESC line H9 cells had a normal female karyotype with 46 chromosomes at metaphase (Fig. 2D). These results strongly suggested that single-cell-state hPSC culture improved transfection efficiency without inducing differentiation or loss of pluripotency.

### Efficient gene editing of single-cell-state hPSC cultures

Efficient gene editing is dependent on the delivery efficiency of gene editing vectors. We used our efficient transfection method to edit the hPSC genome using TALENs. We constructed a Platinum TALEN driven by the EF1α promoter targeting the adenomatous polyposis coli (*APC*) gene and analyzed the efficiency of gene editing using the Cel-1 assay. Electroporation, which is a common delivery method for gene editing,^12–18^ was used as a control.

**FIG. 3.**
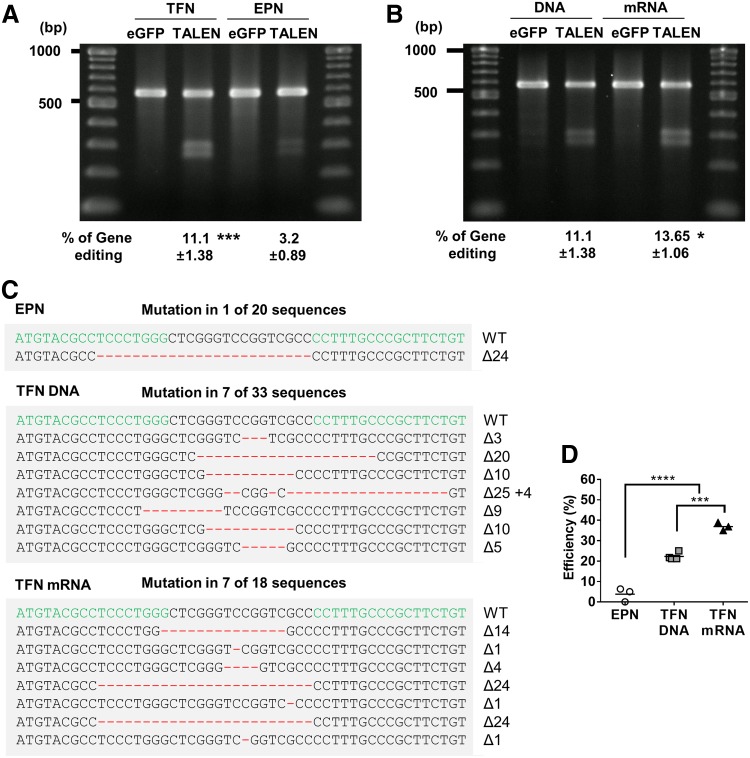
Functional demonstration of the transfection method using TALENs. **(A)** Cel-I assay for TFN and EPN of the TALEN vector targeting the APC gene. PCR products from eGFP-transfected control cells and cells transfected with TALEN loci were analyzed by agarose gel electrophoresis. The efficiency of gene editing is indicated under each guide. Data are shown as the mean ± SD (*n* = 3–4). **(B)** Cel-I assay for transfection of the TALEN vector and mRNA targeting the *APC* gene. PCR products from eGFP-transfected control cells and cells transfected with TALEN loci were analyzed by agarose gel electrophoresis. The efficiency of gene editing is indicated under each guide. Data are shown as the mean ± SD (*n* = 4). **(C)** DNA sequences and frequencies of TALEN-induced mutations in endogenous human genes. The data shown are representative of three to four experiments. For endogenous gene targets, the WT sequence is shown at the top with TALEN target sites highlighted by green. Dashes indicate deletions highlighted by red. The sizes of the insertions (+) or deletions (Δ) are indicated to the right of each mutated site. **(D)** The efficiency of both TALEN vector and mRNA detected by Cel-1 assay. The efficiencies are calculated as the number of mutants identified divided by the total number of sequences analyzed. *p < 0.05, ***p < 0.005, and ****p < 0.001. PCR, polymerase chain reaction; TALENs, transcription activator-like effector nucleases; WT, wild type.

Two days after transfection, we isolated genomic DNA from hPSCs and performed PCR to amplify the *APC* gene. The PCR product was denatured and reannealed, generating a heteroduplex between wild-type and modified amplicons. It was then treated with Cel-1 nucleases to digest heteroduplex DNA. Our efficient transfection method induced mutations more efficiently than electroporation (TFN: 11.1% ± 1.38%, EPN: 3.2 ± 0.89, Fig. 3A). Moreover, we synthesized TALEN mRNA using IVT and transfected it into hPSCs using our method (Fig. 3B). To verify the mutations, we sequenced the target loci. As expected, this experiment revealed characteristic insertion or deletion mutations (indels) at the target gene sites (Fig. 3C). The gene editing efficiency using TALEN mRNA was higher than that of DNA (Fig. 3B, D). These results showed that TALENs increased gene editing efficiency in single-cell-state hPSC cultures.

## Discussion

The purpose of this study was to develop a simple and efficient method of transfecting DNA or mRNA into hPSCs under defined conditions. This is important because the ability to efficiently manipulate the hPSC genome may help to unlock the full potential of hPSCs for use in regenerative medicine and pharmaceutical development and discovery. However, in the past, gene transfer into hPSCs was difficult.^1,4–6,32,33^ In this study, we demonstrated an efficient method for DNA and mRNA transfection into hPSCs. An important feature of this method is its high transfection efficiency, which is achieved by using single-cell-state hPSC cultures, whereas normally, hPSCs form tightly packed colonies that reduce transfection efficiency.^29–31^

Interestingly, our method resulted in heterogeneous eGFP expression in DNA-transfected cells; however, this was not observed after mRNA transfection. The eukaryotic cell nucleus separates the processes of transcription and translation, where eukaryotic transcription occurs in the nucleus. DNA delivery to the nucleus, but not the cytoplasm, is important for DNA transfection. The cells that undergo one round of mitosis after exposure to DNA-liposome complexes are transfected much more efficiently than cells arrested in their cell cycle.^34^ Therefore, eGFP expression heterogeneity after DNA transfection may be due to different cell cycle stages in hPSCs. Effective cell cycle regulation methods may reduce heterogeneous expression.

Although electroporation is a common transfection method for hPSC gene editing, its efficiency is low.^12–16^ Thus, an efficient gene transfer method is needed to stably generate gene-edited cell lines. In this study, we used TALENs for hPSC gene editing. We improved editing efficiency by using a single-cell-state hPSC culture method. Moreover, we compared the gene editing efficiency of TALENs between DNA and mRNA. Our results showed higher efficiency using TALEN mRNA transfection. This result is consistent with the previous report.^35^ We concluded that mRNA transfection is a better method for hPSC gene editing due to increased efficiency and expression homogeneity with no insertional mutagenesis.

Overall, our efficient hPSC transfection method using single-cell-state culture provides an excellent experimental system to investigate the full potential of hPSCs.^36^ We expect that this method may contribute to the fields of hPSC-based regenerative medicine and drug discovery.

## Conclusions

In conclusion, we demonstrated highly efficient transfection and gene editing methods in hPSCs cultured in a single-cell state. We found that transfection of hPSCs maintained in a single-cell state on MATRIX-coated dishes significantly increased the transfection efficiency of both DNA and mRNA compared to the transfection efficiency of cells cultured through conventional methods using electroporation, one of the most efficient methods currently in use. This study also confirms that the transfection efficiency of hPSCs depends on the cell density and that DNA delivery to the nucleus is important for DNA transfection. Furthermore, we showed that single-cell-state culture can improve the efficiency of genome-editing methods. Using TALEN, we achieved more than a threefold increase in the mutation rate of single-cell-state cultured hPSCs compared to that in electroporated hPSCs. The full potential of hPSCs, such as their self-renewal and differentiation capabilities, remains to be understood and further research is needed to fully realize their practical applications. We expect that our findings will advance hPSC research and facilitate their use for human regenerative medicine and drug discovery.

## Supplementary Material

Supplemental data

Supplemental data

Supplemental data

Supplemental data

Supplemental data

## Abbreviations Used

APC: adenomatous polyposis coli
Cas9: CRISPR-associated nuclease 9
CRISPR: clustered regularly interspaced short palindromic repeat
EF1α: human elongation factor 1-alpha 1
eGFP: enhanced green fluorescent protein
EPN: electroporation
FCM: flow cytometry
hESCs: human embryonic stem cells
hiPSCs: human-induced pluripotent stem cells
hPSCs: human pluripotent stem cells
IVT: *in vitro* transcription
MEFs: mouse embryonic fibroblasts
MFI: mean fluorescence intensity
PBS: phosphate-buffered saline
PCR: polymerase chain reaction
TALENs: transcription activator-like effector nucleases
TFN: transfection
VTN: vitronectin
ZFNs: zinc-finger nucleases
