# Supplementary figures and images for: Single-Cell-State Culture of Human Pluripotent Stem Cells Increases Transfection Efficiency
Source: Biores Open Access. 2016 May 1;5(1):127–36. doi: 10.1089/biores.2016.0009 (PMC4876534; doi:10.1089/biores.2016.0009)

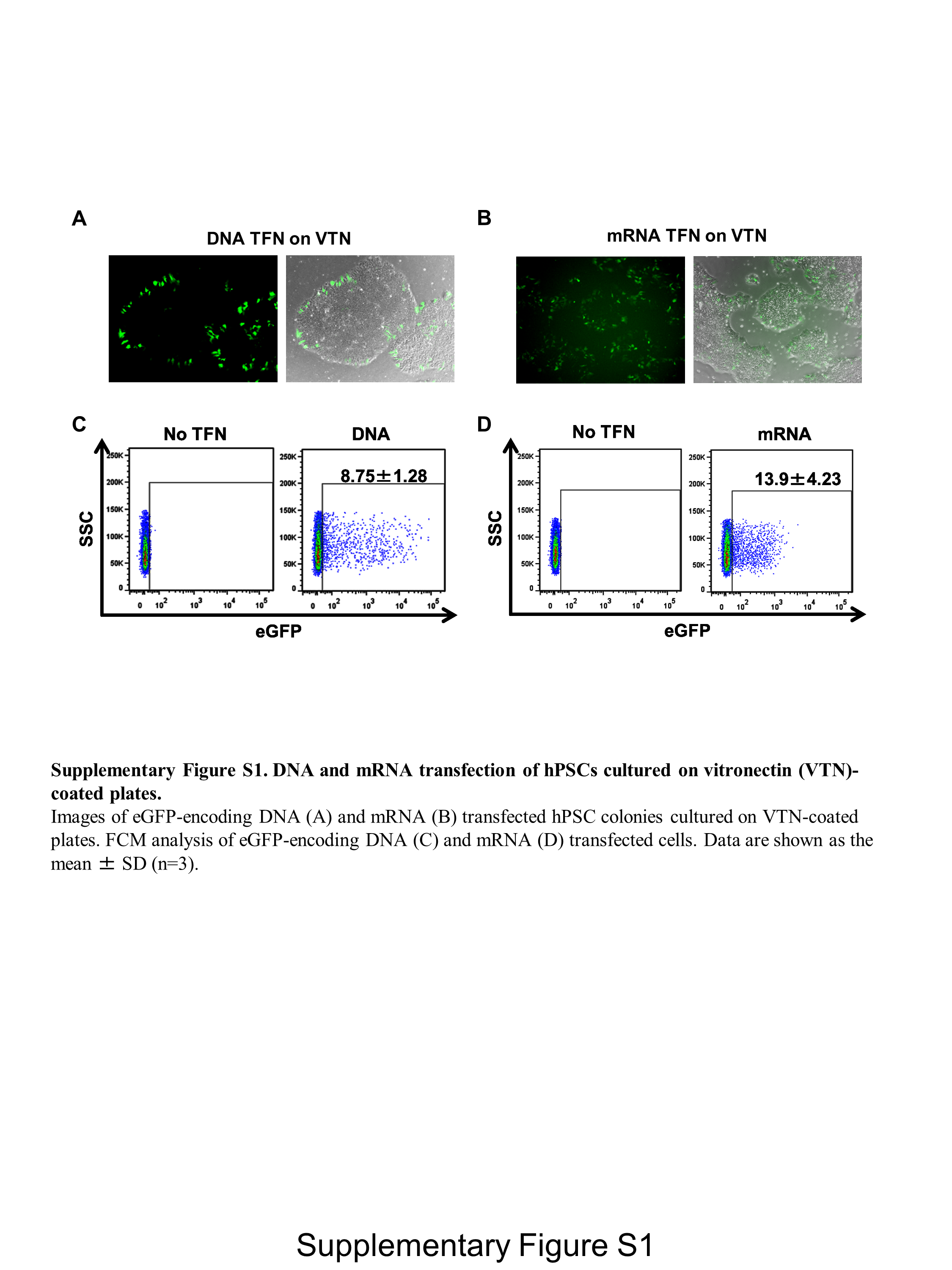

Supplement: Supplemental data [file Supp_Fig1.tif]

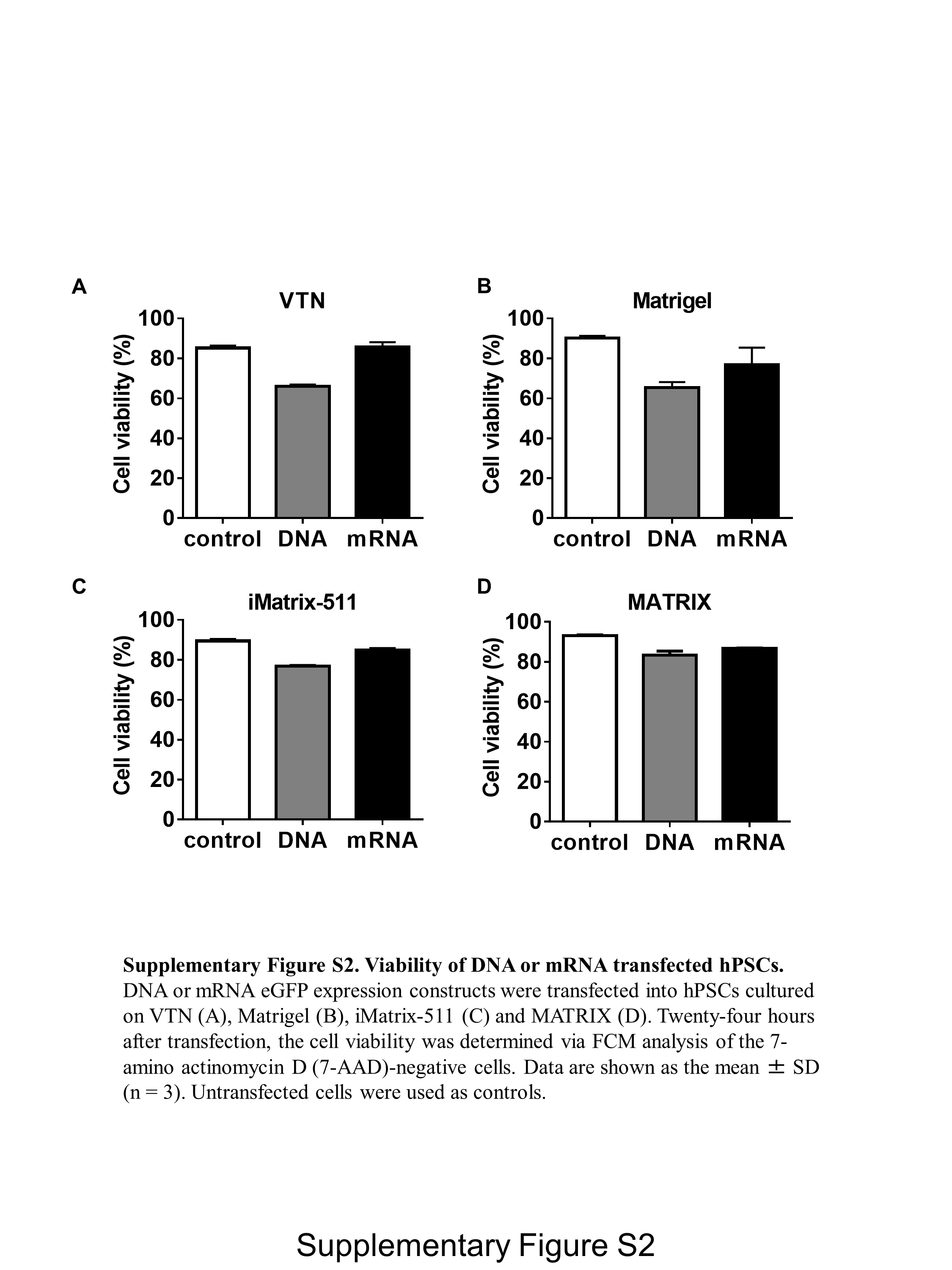

Supplement: Supplemental data [file Supp_Fig2.tif]

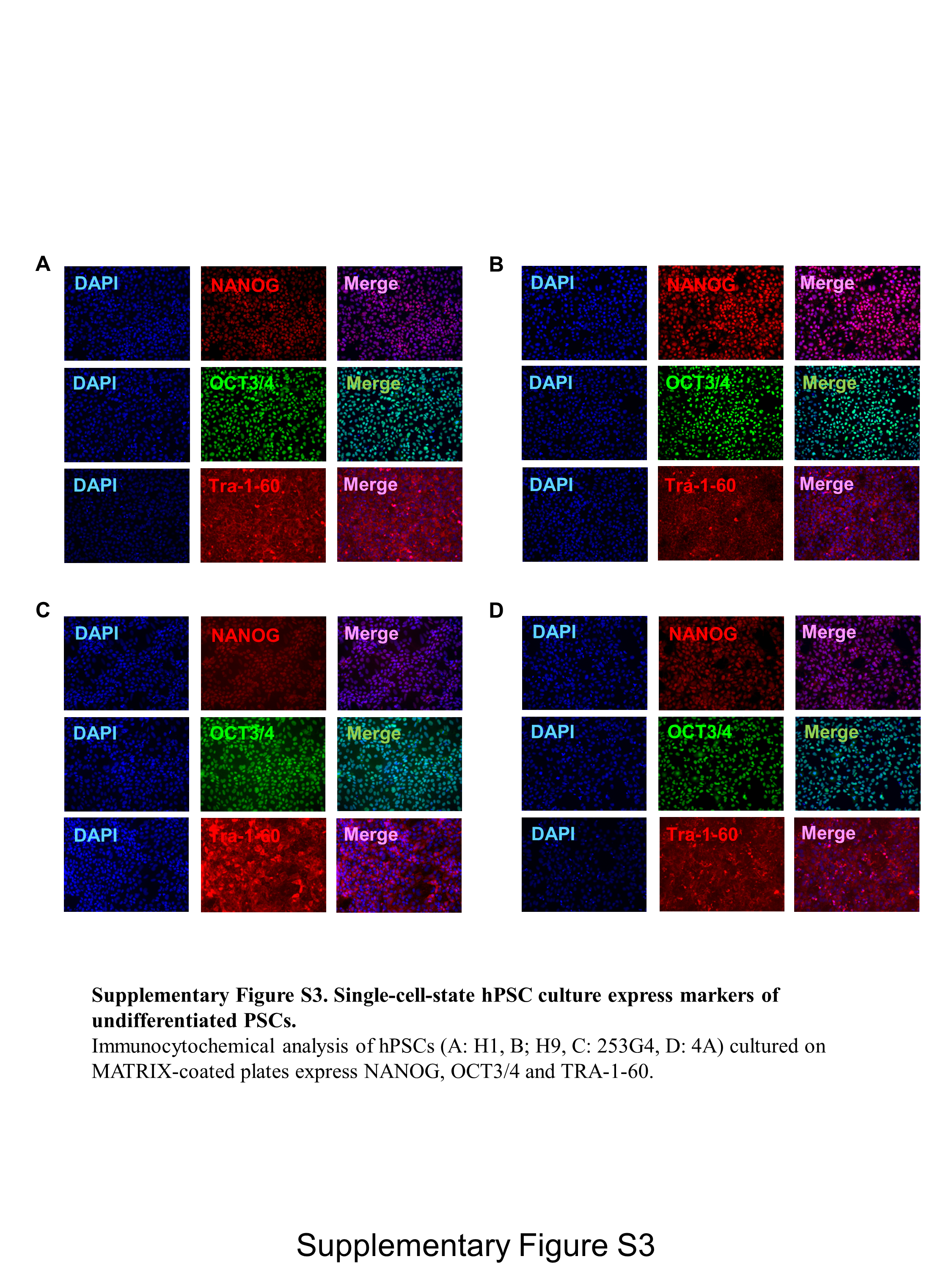

Supplement: Supplemental data [file Supp_Fig3.tif]
